# Supplementary material for: Differences between intrinsic and acquired nucleoside analogue resistance in acute myeloid leukaemia cells
Source: J Exp Clin Cancer Res. 2021 Oct 12;40:317. doi: 10.1186/s13046-021-02093-4 (PMC8507139; doi:10.1186/s13046-021-02093-4)

Supplementary Figure 1. Original uncropped Western Blots

Figure 1A

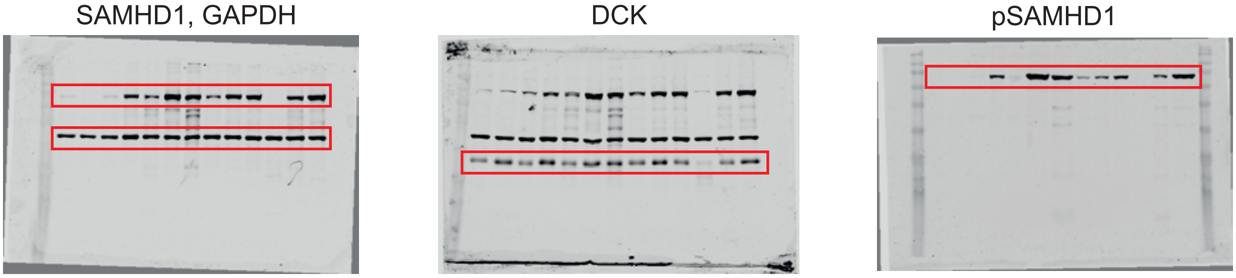

Figure 2C

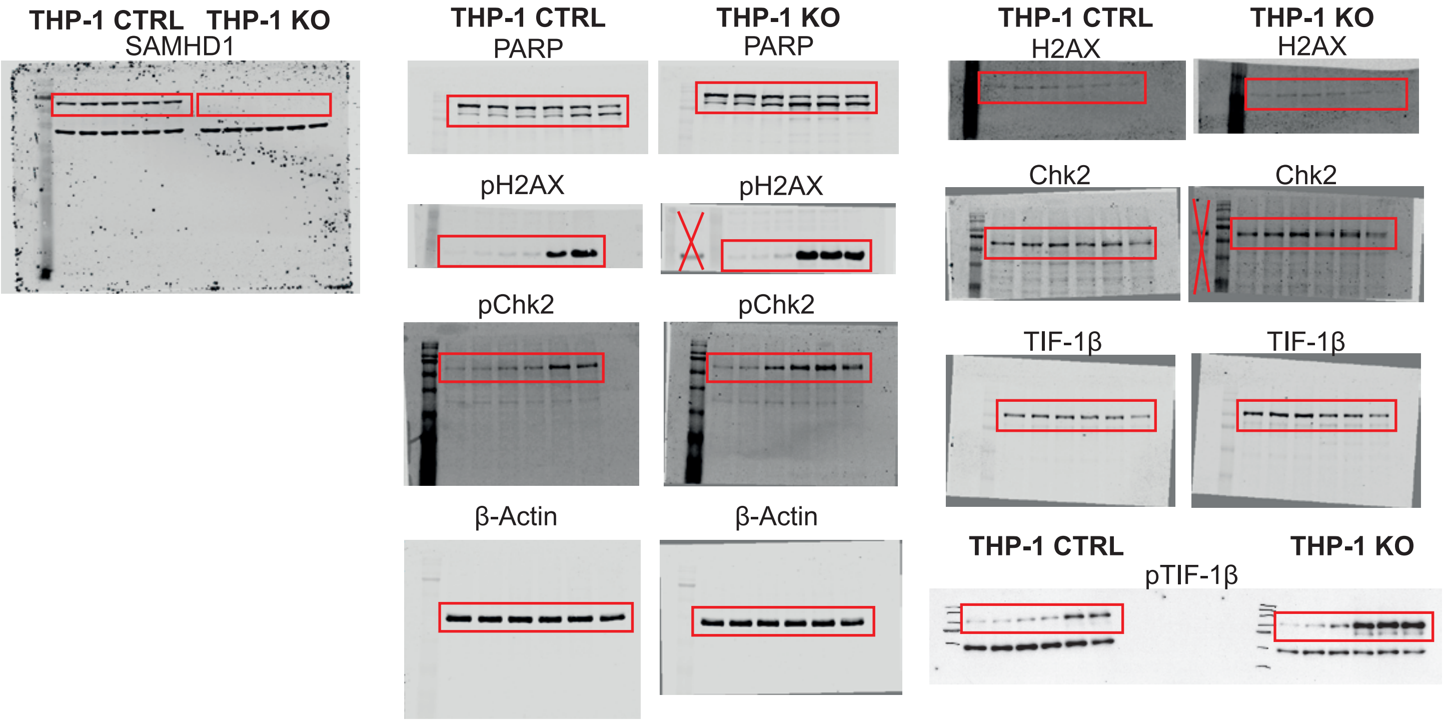

Figure 2E

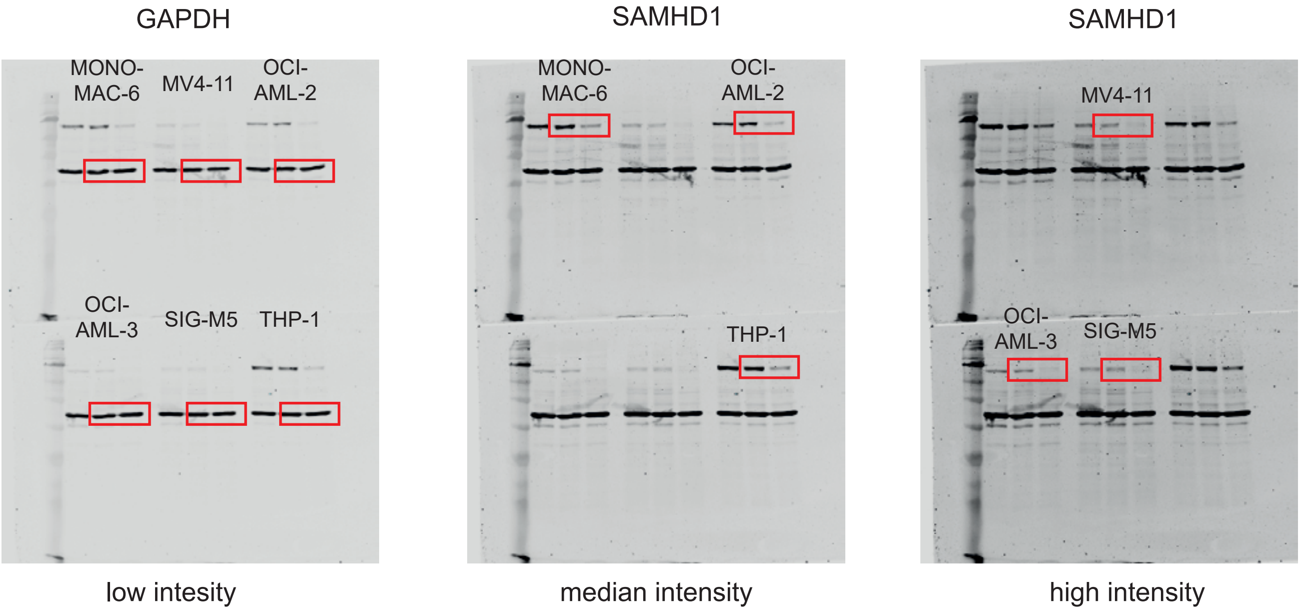

Figure 2G

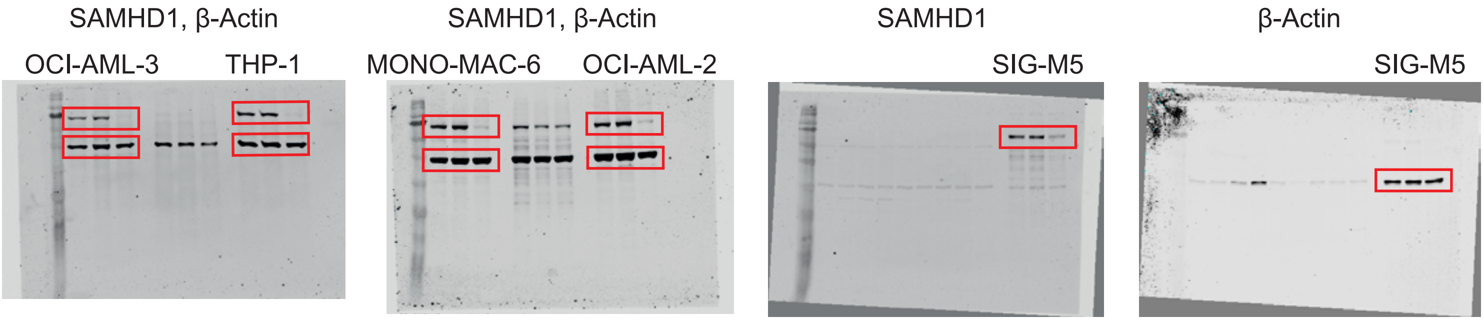

Figure 3D

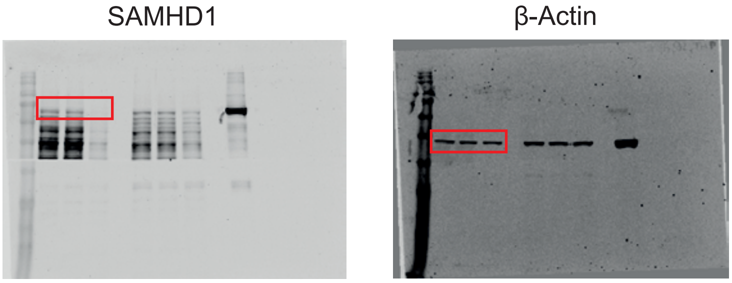

Figure 5C

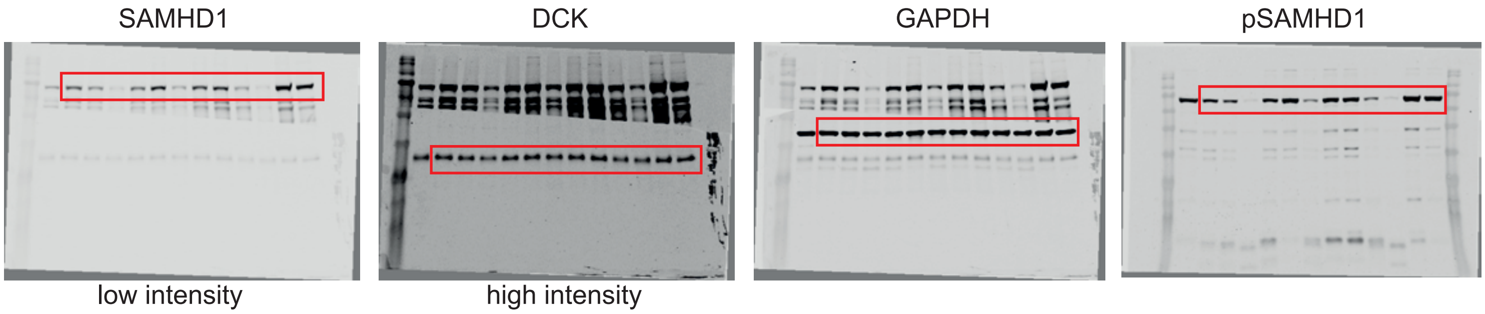

Figure 5F

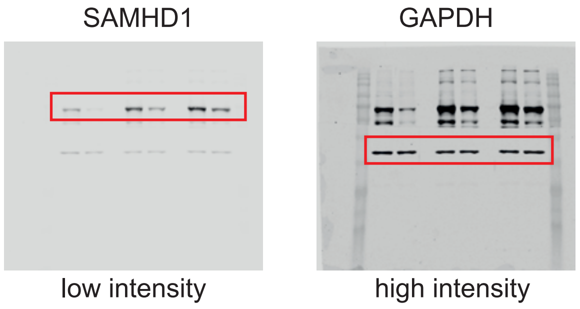

Figure 6A

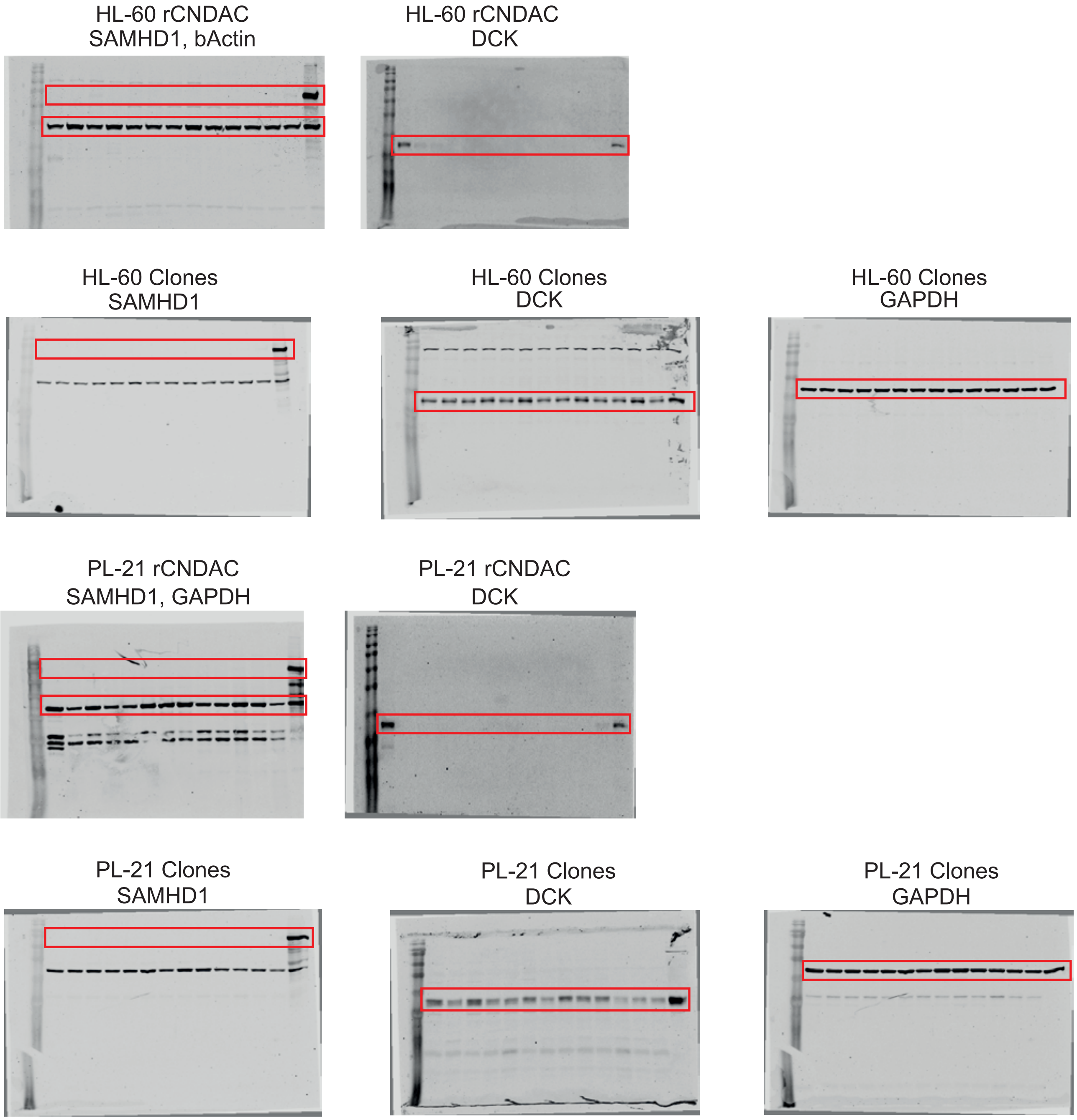

Figure 7A

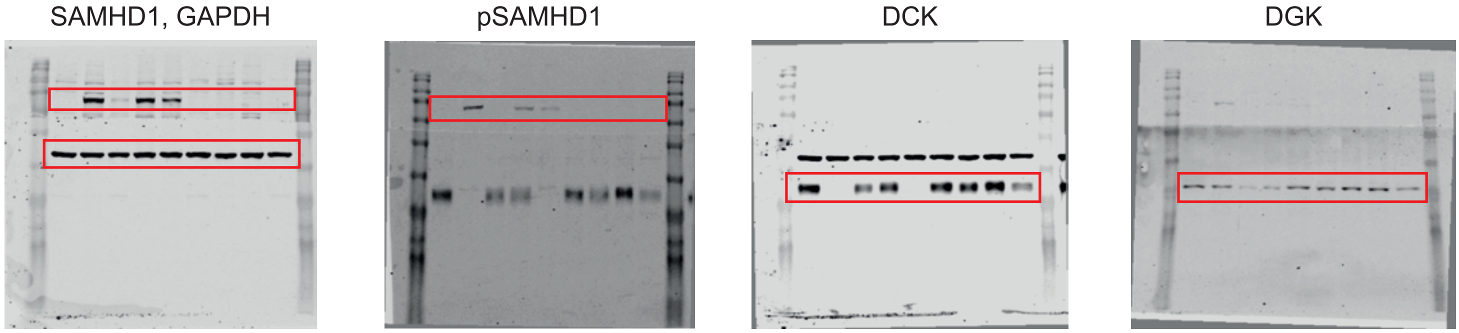

Figure 7D

HL-60 rAraC
SAMHD1, GAPDH

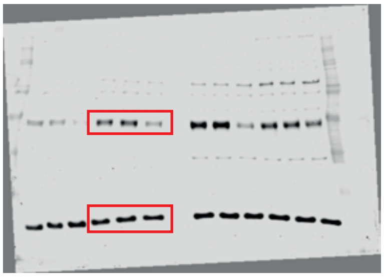

HL-60 rAraG
SAMHD1

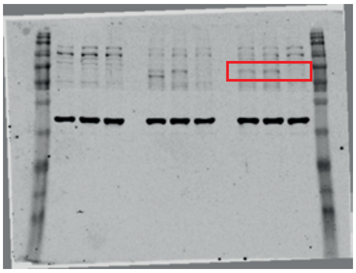

high intensity

HL-60 rAraG
GAPDH

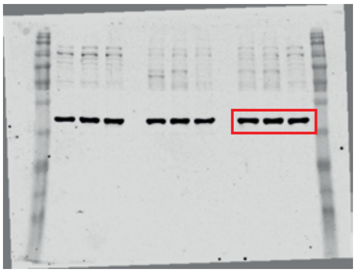

low intensity

HL-60 rAza HL-60 rFluda
SAMHD1

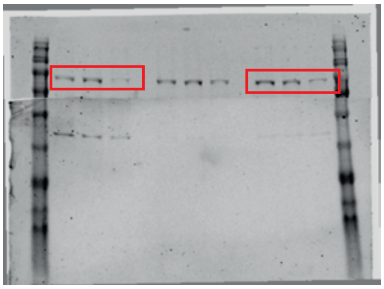

HL-60 rAza HL-60 rFluda
GAPDH

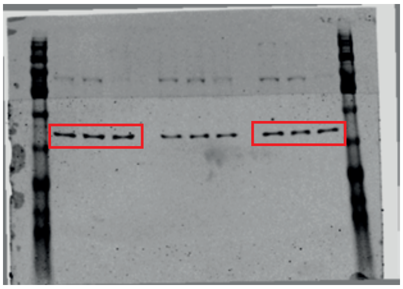

Figure 7E

MV4-11 rAraC pWpi, pWPi DCK
SAMHD1, GAPDH

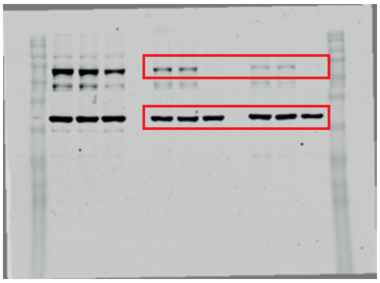

MV4-11 rAraC pWpi, pWPi DCK
pSAMHD1

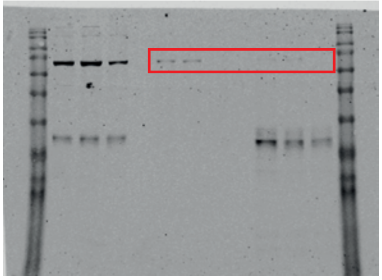

MV4-11 rAraC pWpi, pWPi DCK
DCK

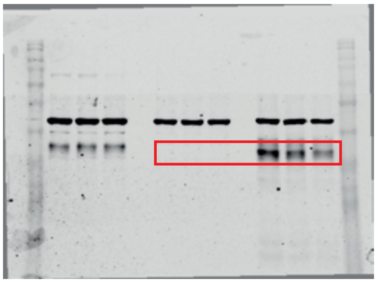

MOLM-13 rAraC pWpi, pWPi DCK
SAMHD1, GAPDH

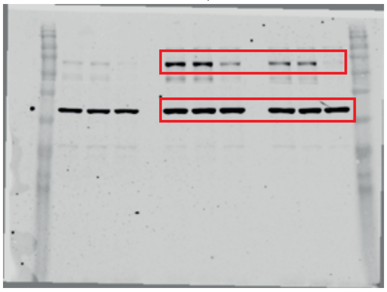

MOLM-13 rAraC pWpi, pWPi DCK
pSAMHD1, DCK

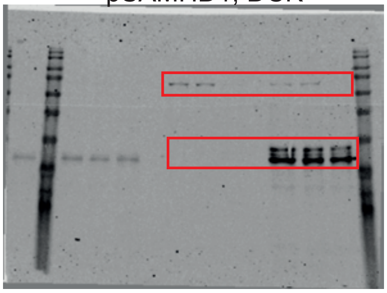

MOLM-13 rAraC pWpi, pWPi DCK
GAPDH
(for pSAMHD1, DCK)

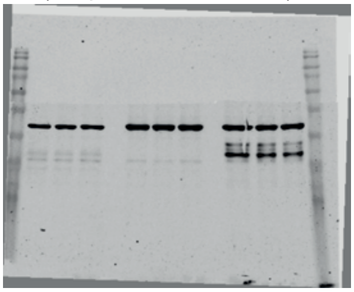

Supplement: Supplementary file 1 — Additional file 1: Supplementary Figure 1. Original uncropped Western Blots. [file 13046_2021_2093_MOESM1_ESM.pdf]
