# Supplementary material for: Differences between intrinsic and acquired nucleoside analogue resistance in acute myeloid leukaemia cells
Source: J Exp Clin Cancer Res. 2021 Oct 12;40:317. doi: 10.1186/s13046-021-02093-4 (PMC8507139; doi:10.1186/s13046-021-02093-4)
Supplement: Supplementary file 2 — Additional file 2: Supplementary Figure 2. Dose-response curves of AML cell lines treated with CNDAC. [file 13046_2021_2093_MOESM2_ESM.pdf]

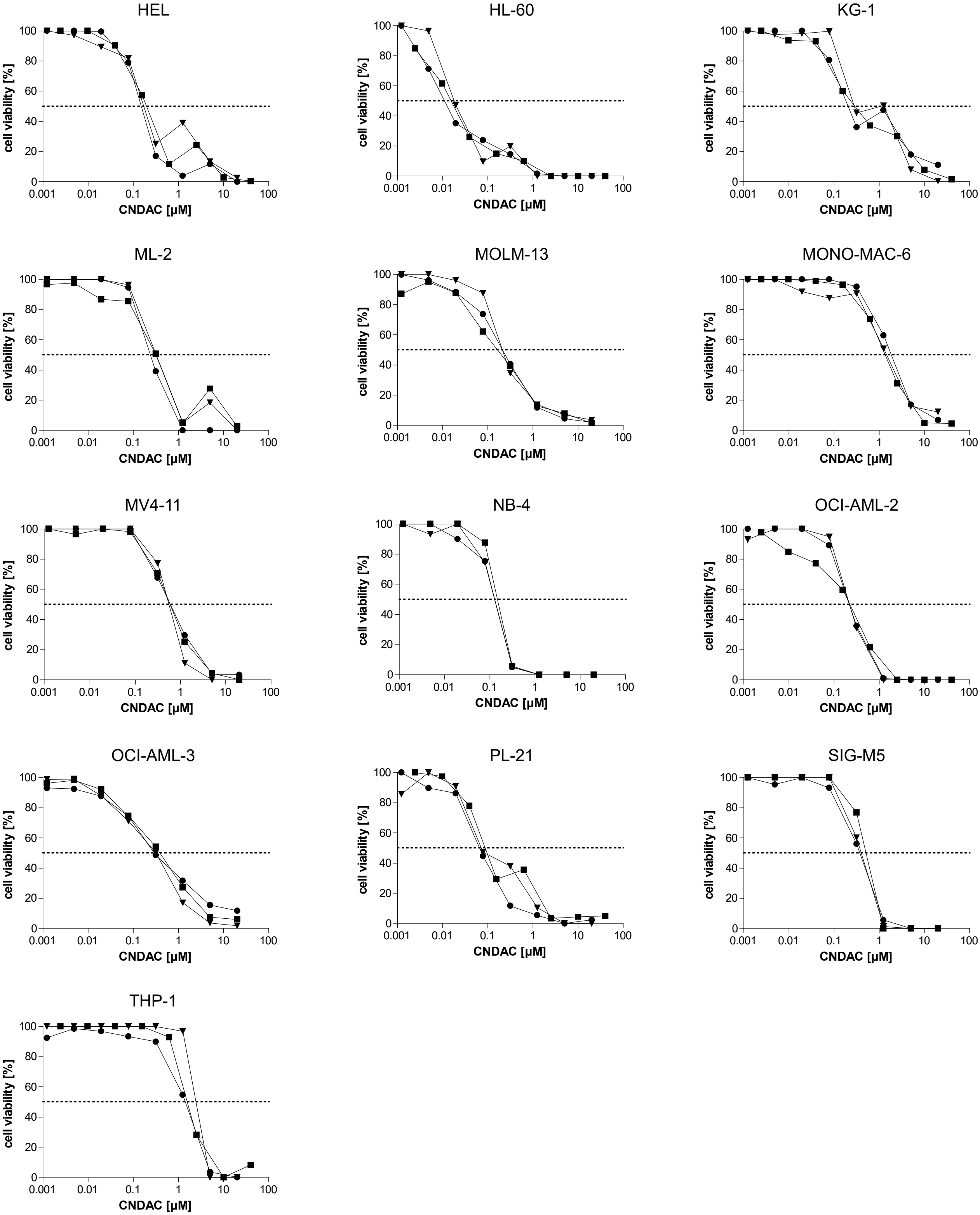

Supplementary Figure 2. Dose-response curves of AML cell lines treated with CNDAC.
13 AML cell lines were treated with different concentrations of CNDAC for 96 hours before viability was quantified by MTT assay. Three independent experiment were performed in three technical replicates and each experiment is shown as one dose-response curve here.
