# Supplementary material for: Differences between intrinsic and acquired nucleoside analogue resistance in acute myeloid leukaemia cells
Source: J Exp Clin Cancer Res. 2021 Oct 12;40:317. doi: 10.1186/s13046-021-02093-4 (PMC8507139; doi:10.1186/s13046-021-02093-4)
Supplement: Supplementary file 3 — Additional file 3: Supplementary Figure 3. SAMHD1 suppression by siRNAs sensitises AML cells to CNDAC. [file 13046_2021_2093_MOESM3_ESM.pdf]

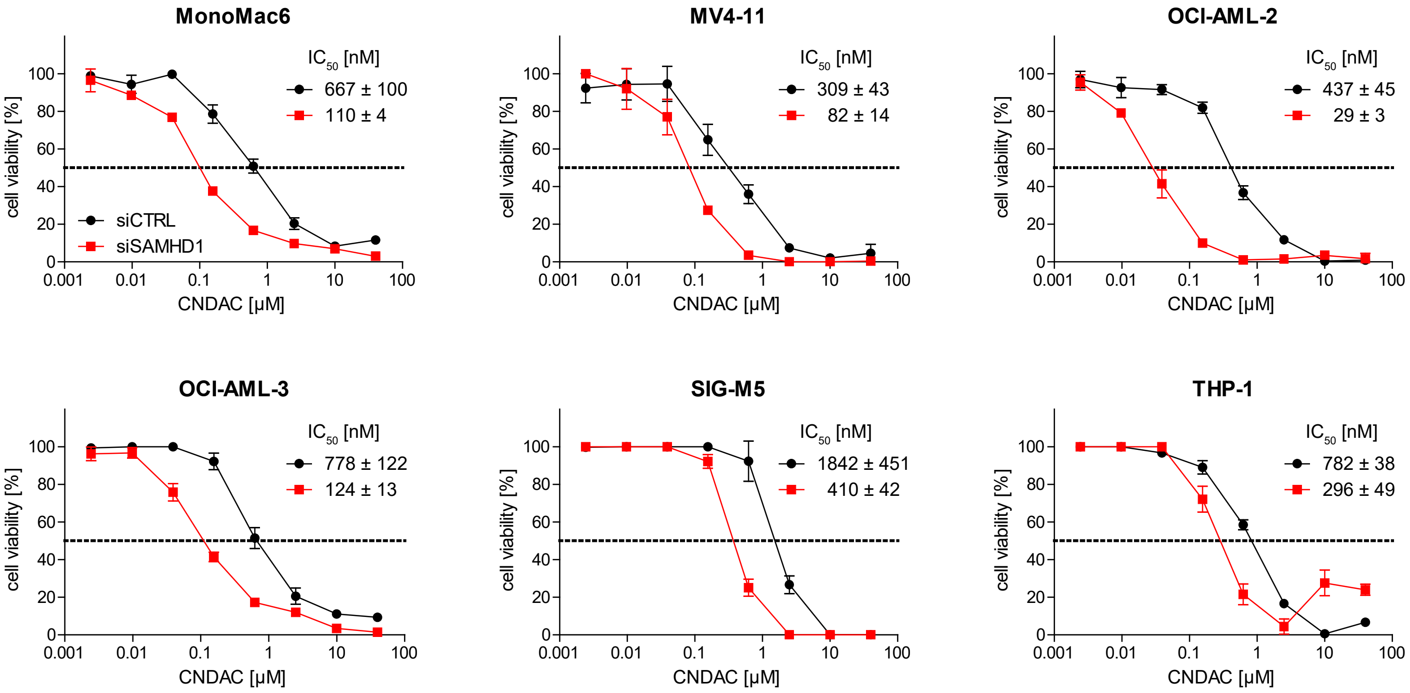

Supplementary Figure 3. SAMHD1 suppression by siRNAs sensitises AML cells to CNDAC.
Dose-response curves of AML cells after transfection with SAMHD1-siRNAs (siSAMHD1) or non targeting control siRNAs (siCTRL) and treatment with CNDAC. 48 hours after transfection, cells where treated with CNDAC and incubated for 96 hours before cell viability was determined by MTT assay. CNDAC concentrations that reduce cell viability by 50% (IC_{50} values) are provided. Symbols represent the means ± SD of three technical replicates of one representative experiment out of three.
