# Supplementary material for: Differences between intrinsic and acquired nucleoside analogue resistance in acute myeloid leukaemia cells
Source: J Exp Clin Cancer Res. 2021 Oct 12;40:317. doi: 10.1186/s13046-021-02093-4 (PMC8507139; doi:10.1186/s13046-021-02093-4)
Supplement: Supplementary file 4 — Additional file 4: Supplementary Figure 4. Analysis of primary AML blasts. [file 13046_2021_2093_MOESM4_ESM.pdf]

A

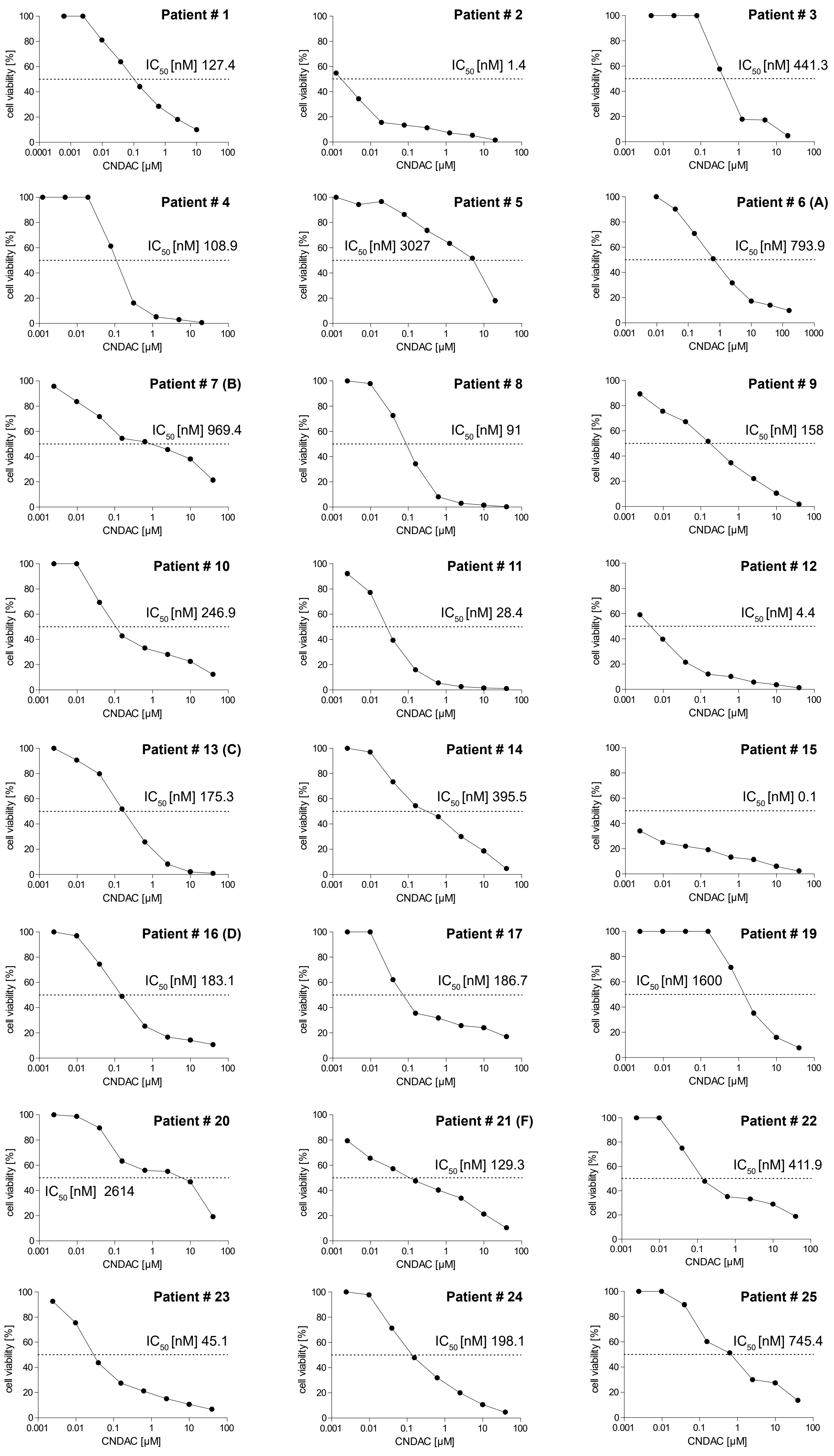

Supplementary Figure 4A. Dose-response curves for CNDAC in primary AML blasts.
Bone marrow-derived AML patient blasts (see also Supplementary Table 4) were treated with different concentrations of CNDAC for 96 hours before viability was quantified by ATP assay. Closed circles represent the means of three technical replicates. CNDAC concentrations that reduce cell viability by 50% (IC$_{50}$ values) are provided.

B

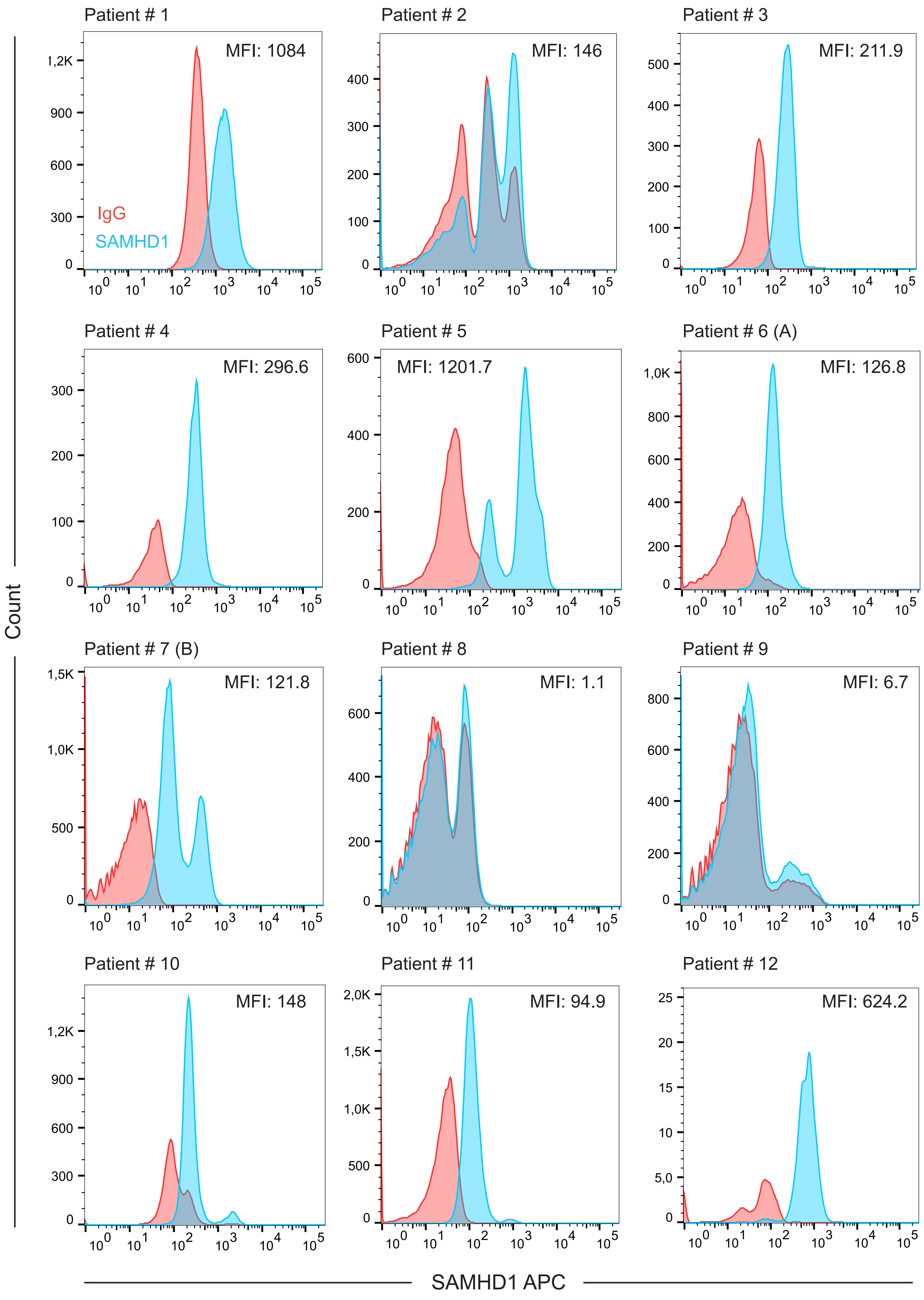
Patient # 1
MFI: 1084
IgG
SAMHD1
Patient # 2
MFI: 146
Patient # 3
MFI: 211.9
Patient # 4
MFI: 296.6
Patient # 5
MFI: 1201.7
Patient # 6 (A)
MFI: 126.8
Patient # 7 (B)
MFI: 121.8
Patient # 8
MFI: 1.1
Patient # 9
MFI: 6.7
Patient # 10
MFI: 148
Patient # 11
MFI: 94.9
Patient # 12
MFI: 624.2
Count
SAMHD1 APC

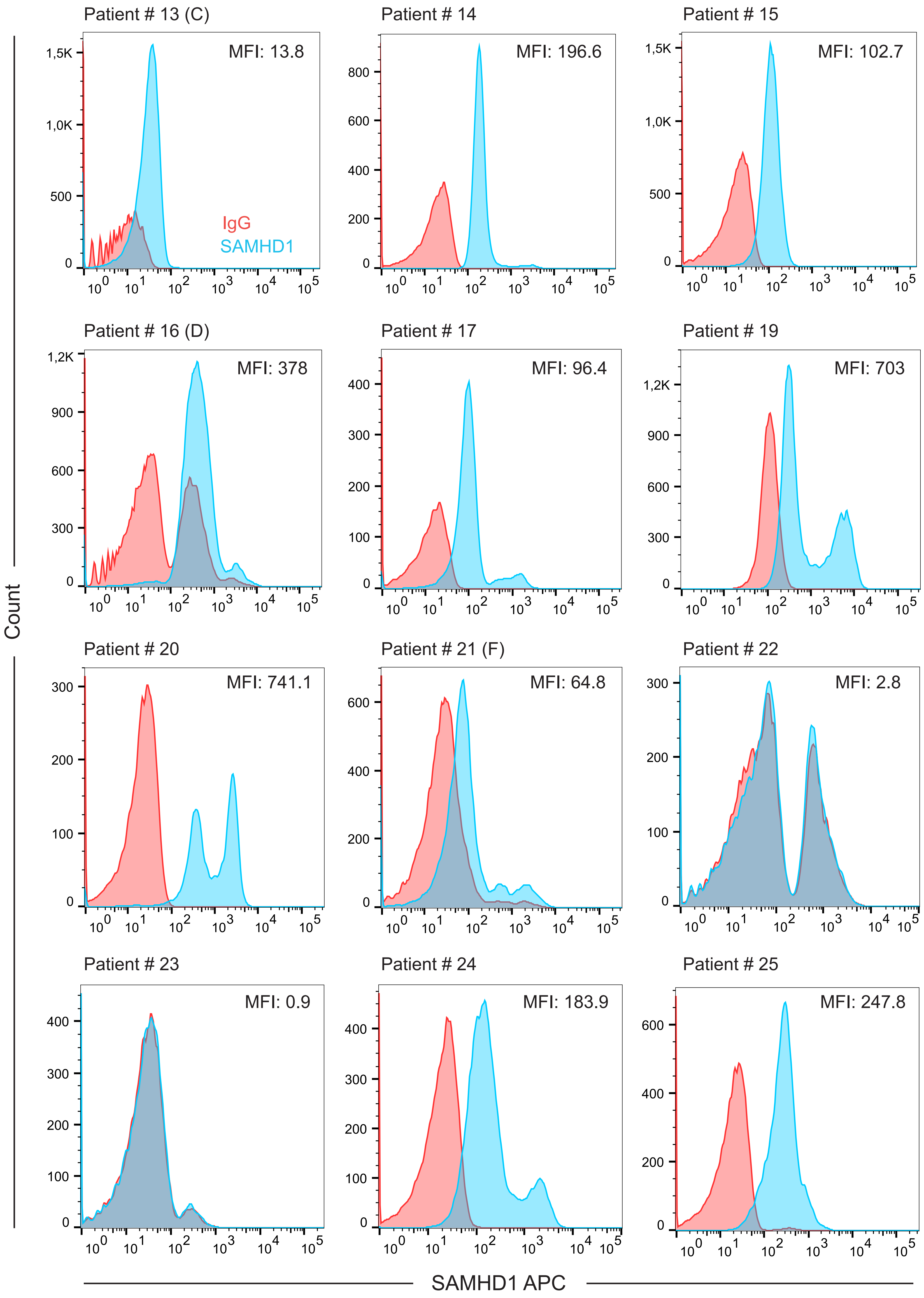

Supplementary Figure 4B. Flow cytometric analysis of primary AML blasts.
AML patient samples (see also Supplementary Table 4) were co-immunostained for CD33, CD34, CD45 (surface markers) and intracellular SAMHD1 and analysed by flow cytometry. Histograms show SAMHD1 expression (blue) and the isotype control staining (IgG, red). To determine the mean fluorescence intensity (MFI) for SAMHD1, the geometric mean for IgG was subtracted from the geometric mean for SAMHD1.
