# Supplementary material for: Differences between intrinsic and acquired nucleoside analogue resistance in acute myeloid leukaemia cells
Source: J Exp Clin Cancer Res. 2021 Oct 12;40:317. doi: 10.1186/s13046-021-02093-4 (PMC8507139; doi:10.1186/s13046-021-02093-4)
Supplement: Supplementary file 5 — Additional file 5: Supplementary Figure 5. SAMHD1 suppression by VPX-VLPs sensitises primary AML blasts to CNDAC. [file 13046_2021_2093_MOESM5_ESM.pdf]

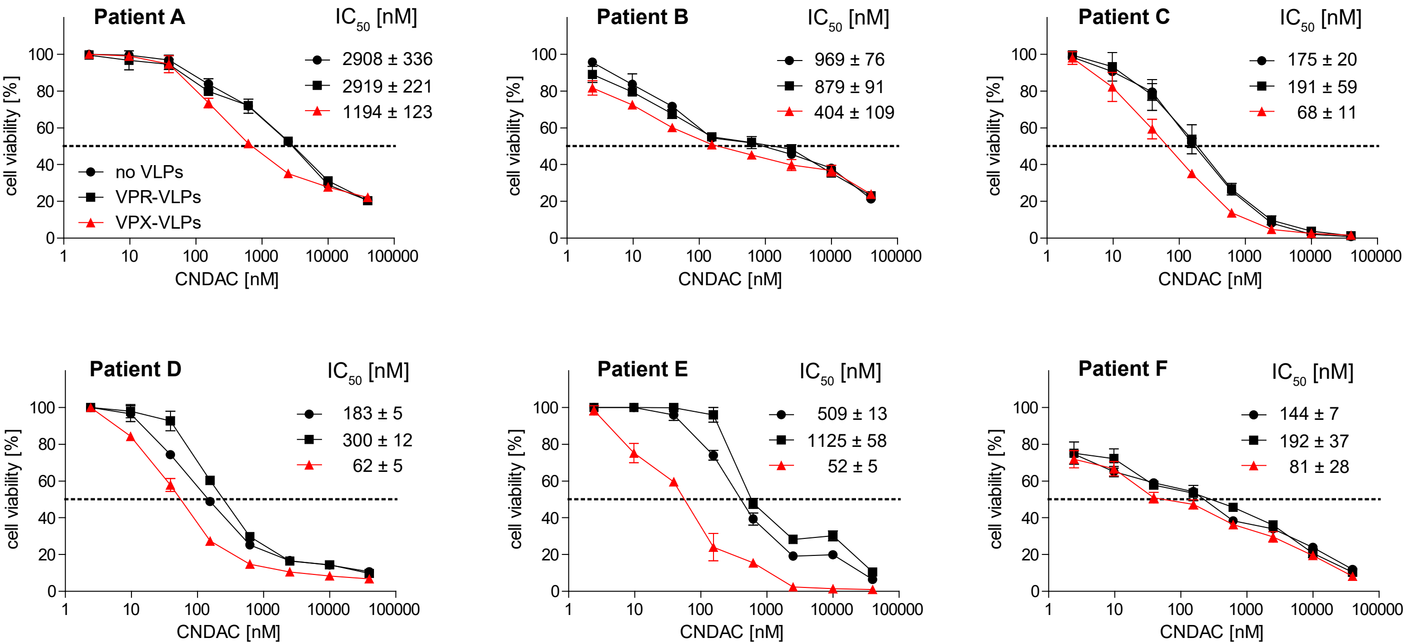

Supplementary Figure 5. SAMHD1 suppression by VPX-VLPs sensitises primary AML blasts to CNDAC.
Dose-response curves of bone-marrow-derived leukaemic blasts from six therapy-naïve AML patients (see also Supplementary Table 4) either pre-treated with VPX virus-like particles (VPX-VLPs, cause SAMHD1 depletion), VPR virus-like particles (VPR-VLPs, negative control) or left untreated. After 24 hours of pre-treatment, different concentrations of CNDAC were added and after additional 96 hours cell viability was determined by ATP assay. CNDAC concentrations that reduce cell viability by 50% (IC_{50} values) are provided. Symbols represent means ± SD from three technical replicates.
