# Supplementary material for: Differences between intrinsic and acquired nucleoside analogue resistance in acute myeloid leukaemia cells
Source: J Exp Clin Cancer Res. 2021 Oct 12;40:317. doi: 10.1186/s13046-021-02093-4 (PMC8507139; doi:10.1186/s13046-021-02093-4)
Supplement: Supplementary file 6 — Additional file 6: Supplementary Figure 6. Illustration of SAMHD1 homotetramerisation and the role of CNDAC-TP. [file 13046_2021_2093_MOESM6_ESM.pdf]

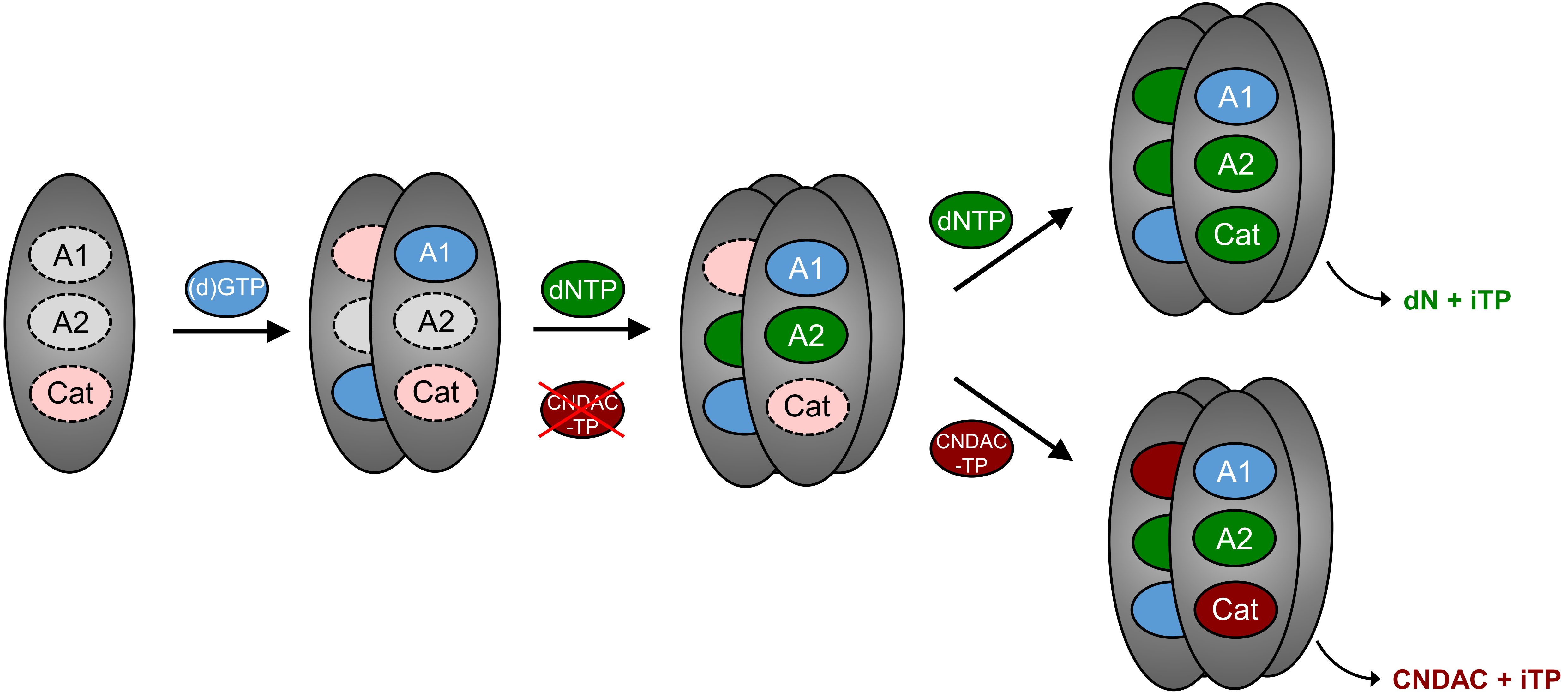

Supplementary Figure 6. Illustration of SAMHD1 homotetramerization and the role of CNDAC-TP.
SAMHD1 monomers form homodimers after binding of GTP (or dGTP) to allosteric site 1 (A1). Binding of any canonical dNTP, or some triphosphorylated deoxyribose-based nucleoside analogues such as Cladribine-TP and Decitabine-TP to allosteric site 2 (A2) enable homotetramerization and therefore activation of SAMHD1. The large nitrile group of CNDAC-TP prevents binding to the A2 site. Active SAMHD1 homotetramers act as dNTP triphosphohydrolase after binding of dNTPs to the catalytic site (Cat) and cleave physiological dNTPs into deoxyribonucleotides and inorganic triphosphate. SAMHD1 is also able to inactivate the triphosphorylated forms of some anti-cancer nucleoside analogues like cytarabine, decitabine and nelarabine. As the catalytic site of SAMHD1 is less restrictive than the A2 site, CNDAC-TP fits into the catalytic pocket and can be inactivated by hydrolysation into CNDAC and inorganic triphosphate.
