# Supplementary material for: Differences between intrinsic and acquired nucleoside analogue resistance in acute myeloid leukaemia cells
Source: J Exp Clin Cancer Res. 2021 Oct 12;40:317. doi: 10.1186/s13046-021-02093-4 (PMC8507139; doi:10.1186/s13046-021-02093-4)
Supplement: Supplementary file 7 — Additional file 7: Supplementary Figure 7. Dose-response curves of MV4–11 clones treated with CNDAC. [file 13046_2021_2093_MOESM7_ESM.pdf]

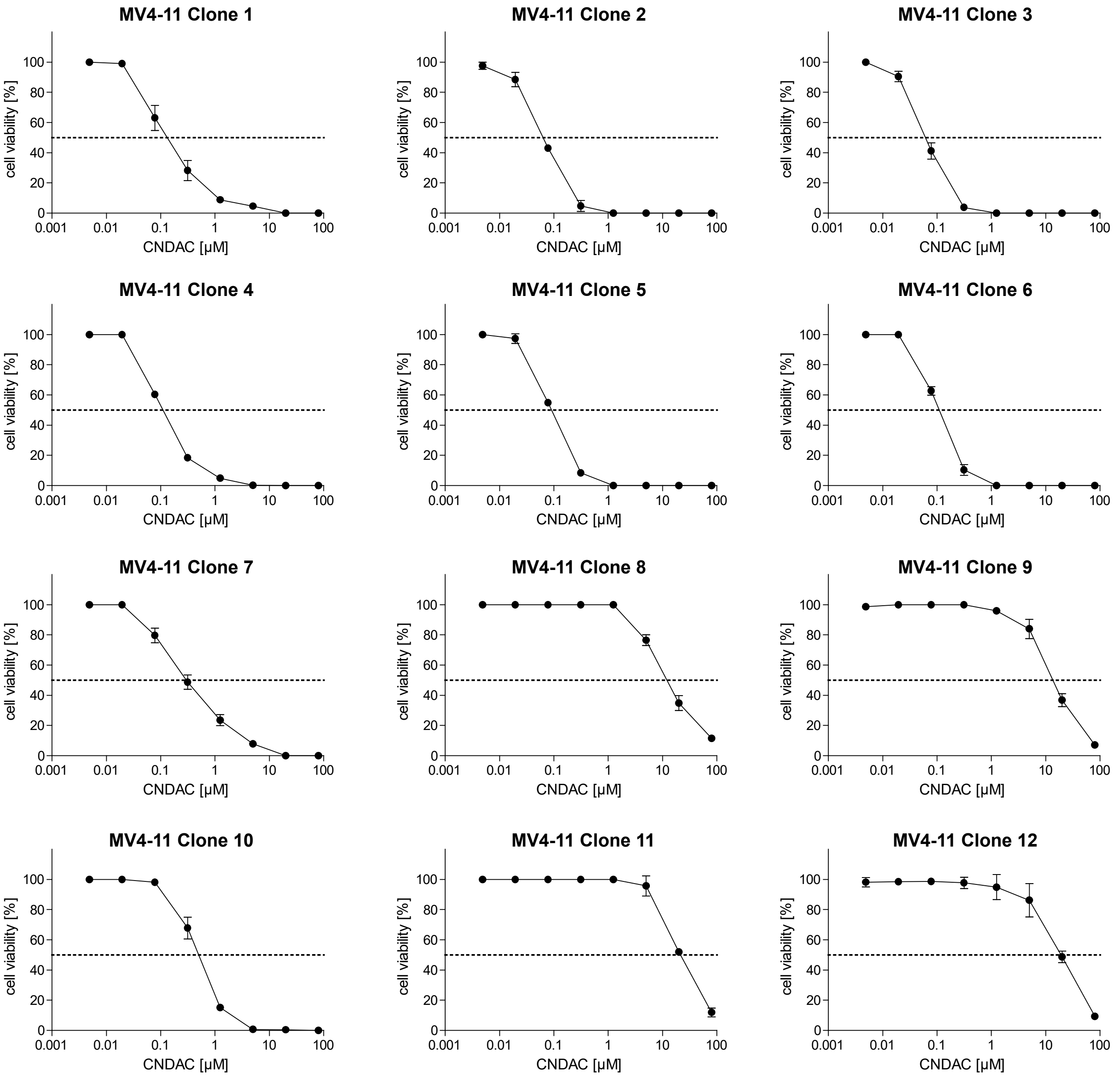

Supplementary Figure 7. Dose-response curves of MV4-11 clones treated with CNDAC. MV4-11 single cell-derived clones were established by limited dilution. Cells were treated with different concentrations of CNDAC for 96 hours before viability was quantified by MTT assay. Closed circles and error bars show means ± SD from three independent experiments, each performed in three technical replicates.
