# Supplementary material for: Differences between intrinsic and acquired nucleoside analogue resistance in acute myeloid leukaemia cells
Source: J Exp Clin Cancer Res. 2021 Oct 12;40:317. doi: 10.1186/s13046-021-02093-4 (PMC8507139; doi:10.1186/s13046-021-02093-4)
Supplement: Supplementary file 8 — Additional file 8: Supplementary Figure 8. SAMHD1 suppression by siRNAs sensitises MV4–11 clones to CNDAC, but not to Daunorubicin. [file 13046_2021_2093_MOESM8_ESM.pdf]

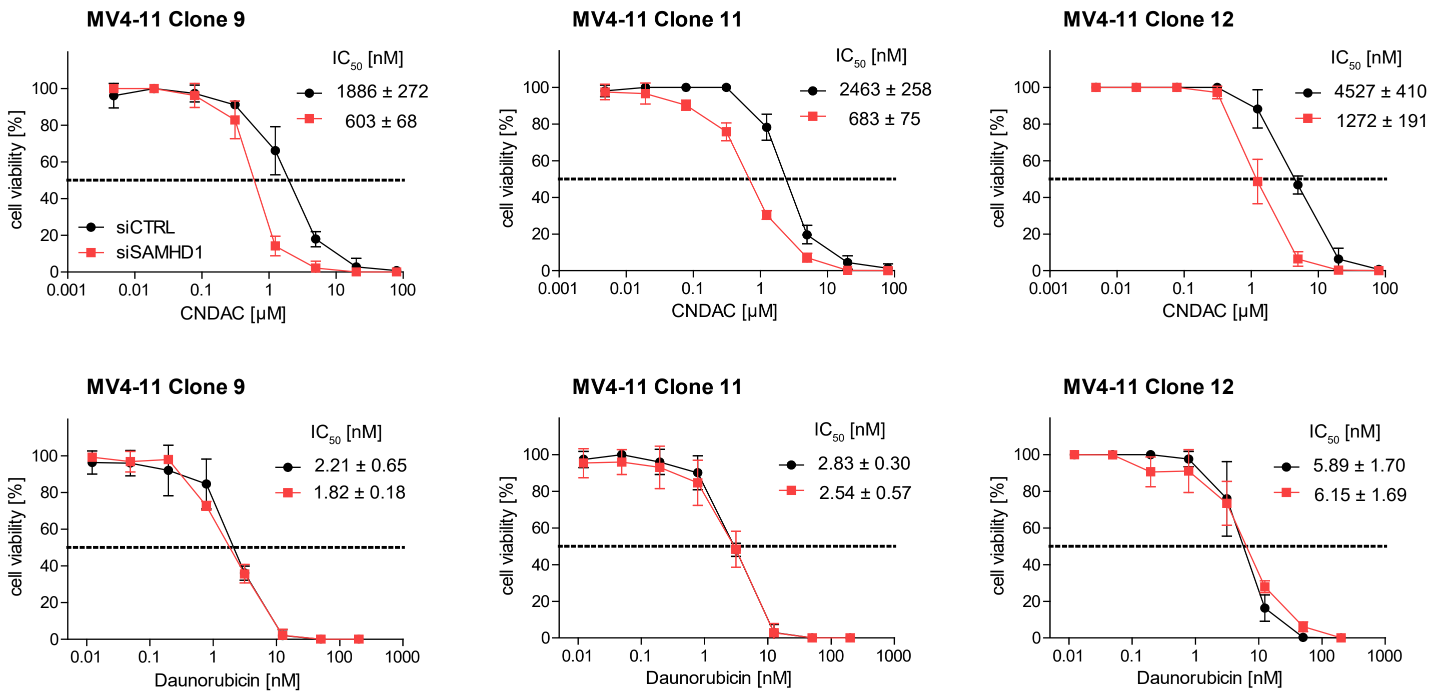

Supplementary Figure 8. SAMHD1 suppression by siRNAs sensitises MV4-11 clones to CNDAC, but not to Daunorubicin. Dose-response curves of MV4-11 single cell-derived clones after transfection with SAMHD1-siRNAs (siSAMHD1) or non targeting control siRNAs (siCTRL) and treatment with CNDAC or Daunorubicin. 48 hours after transfection, cells where treated with CNDAC or Daunorubicin and incubated for 96 hours before cell viability was determined by MTT assay. CNDAC concentrations that reduce cell viability by 50% (IC50 values) are provided. Symbols and error bars represent means ± SD of three technical replicates of one representative experiment out of three.
