# Supplementary material for: Differences between intrinsic and acquired nucleoside analogue resistance in acute myeloid leukaemia cells
Source: J Exp Clin Cancer Res. 2021 Oct 12;40:317. doi: 10.1186/s13046-021-02093-4 (PMC8507139; doi:10.1186/s13046-021-02093-4)
Supplement: Supplementary file 9 — Additional file 9: Supplementary Figure 9. Dose-response curves of drug resistant HL-60 cells. [file 13046_2021_2093_MOESM9_ESM.pdf]

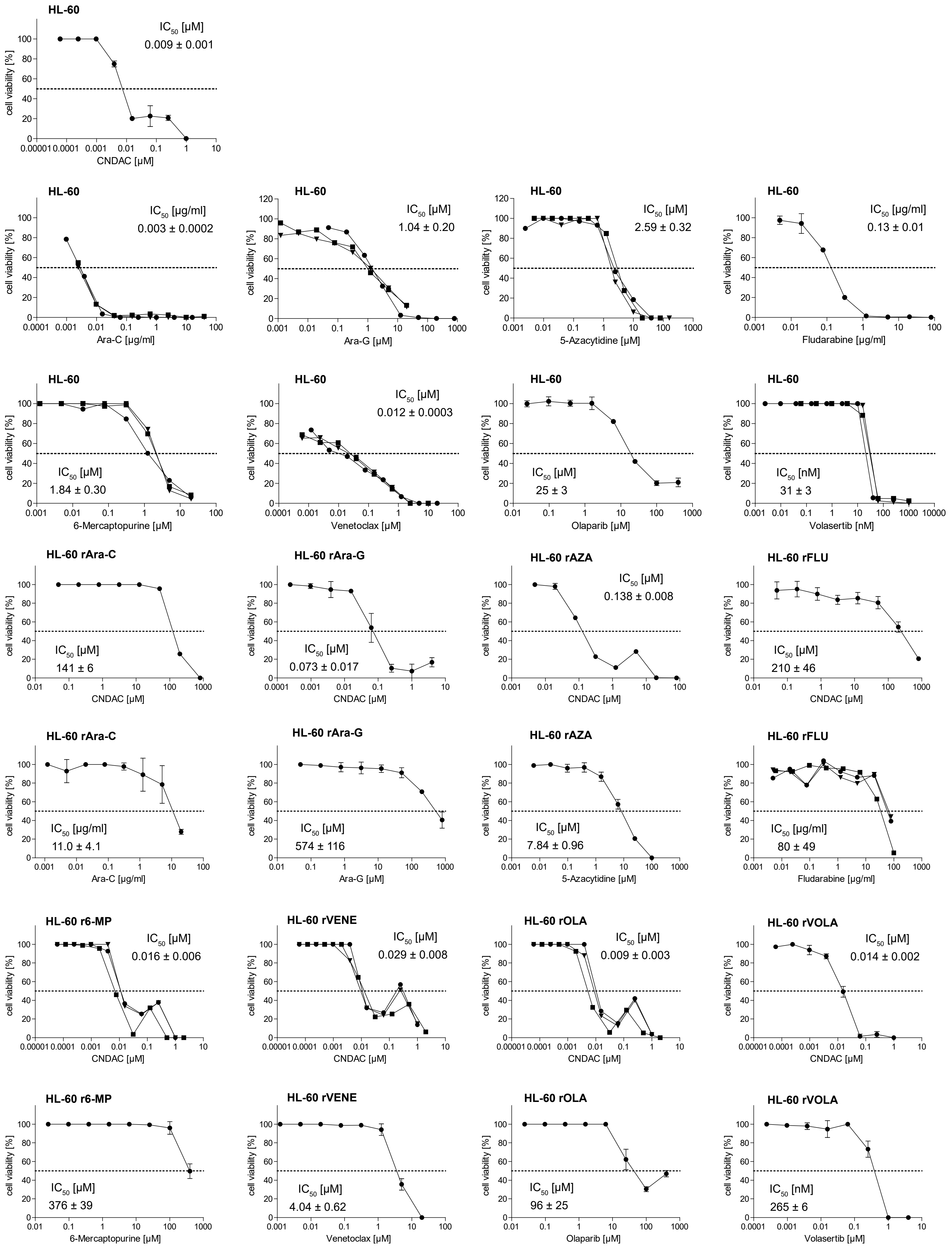

Supplementary Figure 9. Dose-response curves of drug resistant HL-60 cells.
HL-60 parental cells and HL-60 cells resistant to the drugs Cytarabine (Ara-C), Ara-G, 5-Azacytidine (AZA), Fludarabine (FLU), 6-Mercaptopurine (6-MP), Venetoclax (VENE), Olaparib (OLA) and Volasertib (VOLA) were treated with different concentrations of CNDAC or the respective drug they were adapted to (to prove successful adaptation). After 96 hours cell viability was determined by MTT assay. Three independent experiments, each performed in three technical replicates are either shown as means ± SD or, if the starting concentrations of the three experiments differed, each experiment as one dose-response curve. Drug concentrations that reduce cell viability by 50% (IC50s) are provided.
