# Supplementary material for: Differences between intrinsic and acquired nucleoside analogue resistance in acute myeloid leukaemia cells
Source: J Exp Clin Cancer Res. 2021 Oct 12;40:317. doi: 10.1186/s13046-021-02093-4 (PMC8507139; doi:10.1186/s13046-021-02093-4)
Supplement: Supplementary file 10 — Additional file 10: Supplementary Table 1. CNDAC concentrations that reduce AML cell viability by 50% (IC50), relative SAMHD1 protein levels, quantified using near-infrared Western blot images to determine the ratio SAMHD1/ GAPDH, and CNDAC-triphosphate levels determined by LC-MS/MS. [file 13046_2021_2093_MOESM10_ESM.pdf]

Supplementary Table 1. CNDAC concentrations that reduce AML cell viability by 50% (IC_{50}), relative SAMHD1 protein levels, quantified using near-infrared Western blot images to determine the ratio SAMHD1/ GAPDH, and CNDAC-triphosphate levels determined by LC-MS/MS.

	IC_{50} CNDAC [nM]	rel. SAMHD1 protein level	CNDAC-TP [cps x 10^5]
HEL	169 ± 17	0.0096 ± 0.0046	7.36 ± 0.82
HL-60	15 ± 3	0.0002 ± 0.0002	7.17 ± 0.73
KG-1	266 ± 39	0.0143 ± 0.0087	1.74 ± 0.13
ML-2	298 ± 27	0.1043 ± 0.0470	0.35 ± 0.04
MOLM-13	201 ± 26	0.0989 ± 0.0256	0.31 ± 0.03
MONO-MAC-6	1524 ± 179	0.9465 ± 0.2152	0.44 ± 0.07
MV4-11	565 ± 30	0.5404 ± 0.1500	0.00 ± 0.00
NB4	122 ± 11	0.0788 ± 0.0151	0.57 ± 0.03
OCI-AML-2	230 ± 11	0.4212 ± 0.0936	0.00 ± 0.00
OCI-AML-3	331 ± 39	0.3442 ± 0.0897	0.20 ± 0.02
PL-21	77 ± 9	0.0004 ± 0.0003	11.07 ± 0.68
SIG-M5	351 ± 2	0.2083 ± 0.0330	0.09 ± 0.07
THP-1	1598 ± 177	1.0000 ± 0.0000	0.00 ± 0.00
