# Supplementary material for: Differences between intrinsic and acquired nucleoside analogue resistance in acute myeloid leukaemia cells
Source: J Exp Clin Cancer Res. 2021 Oct 12;40:317. doi: 10.1186/s13046-021-02093-4 (PMC8507139; doi:10.1186/s13046-021-02093-4)
Supplement: Supplementary file 11 — Additional file 11: Supplementary Table 2. CNDAC concentrations that reduce ALL cell line viability by 50% (IC50), relative SAMHD1 protein levels quantified using near-infrared Western blot images to determine the ratio SAMHD1/ GAPDH relative to the positive control THP-1, and CNDAC-triphosphate levels determined by LC-MS/MS. [file 13046_2021_2093_MOESM11_ESM.pdf]

**Supplementary Table 2.** CNDAC concentrations that reduce ALL cell line viability by 50% ($IC_{50}$), relative SAMHD1 protein levels quantified using near-infrared Western blot images to determine the ratio SAMHD1/ GAPDH relative to the positive control THP-1, and CNDAC-triphosphate levels determined by LC-MS/MS.

| **T-ALL** | | $IC_{50}$ CNDAC [nM] | rel. SAMHD1 protein level | CNDAC-TP [cps x $10^6$] |
|---|---|---|---|---|
| | ALL-SIL | 31.7 ± 6.5 | 0.0110 ± 0.0013 | 3.96 ± 0.30 |
| | CCRF-CEM | 41.2 ± 0.5 | 0.0016 ± 0.0009 | 0.81 ± 0.09 |
| | CTV-1 | 30.6 ± 3.8 | 0.0018 ± 0.0006 | 1.76 ± 0.10 |
| | HSB-2 | 27.0 ± 1.1 | 0.0026 ± 0.0004 | 0.21 ± 0.19 |
| | JJ-HAN | 53.3 ± 8.8 | 0.0035 ± 0.0010 | 1.03 ± 0.03 |
| | JURKAT | 191.1 ± 32.5 | 0.0011 ± 0.0004 | 1.22 ± 0.11 |
| | KE-37 | 41.4 ± 4.5 | 0.0036 ± 0.0022 | 1.50 ± 0.09 |
| | MOLT-4 | 8.7 ± 0.3 | 0.0011 ± 0.0006 | 1.06 ± 0.08 |
| | MOLT-16 | 27.7 ± 4.0 | 0.1683 ± 0.0684 | 0.29 ± 0.04 |
| | P12-ICHIKAWA | 11.8 ± 1.4 | 0.0018 ± 0.0001 | 1.31 ± 0.27 |
| | RPMI-8402 | 16.3 ± 1.1 | 0.0013 ± 0.0010 | 1.64 ± 0.26 |
| **B-ALL** | | | | |
| | 697 | 7.1 ± 0.2 | 0.0155 ± 0.0141 | 1.98 ± 0.09 |
| | BALL-1 | 41.5 ± 4.0 | 0.1500 ± 0.0877 | 0.34 ± 0.22 |
| | GRANTA-452 | 32.1 ± 2.8 | 0.0695 ± 0.0284 | 0.28 ± 0.02 |
| | HAL-01 | 26.0 ± 2.0 | 0.2271 ± 0.0749 | 0.21 ± 0.03 |
| | KARPAS-231 | 69.6 ± 2.5 | 0.2446 ± 0.1520 | 0.13 ± 0.02 |
| | MHH-CALL-4 | 158.4 ± 3.0 | 0.1030 ± 0.0606 | 0.21 ± 0.01 |
| | MN-60 | 865.9 ± 4.3 | 0.4813 ± 0.2562 | 0.13 ± 0.00 |
| | NALM-6 | 32.6 ± 4.9 | 0.0357 ± 0.0242 | 0.88 ± 0.08 |
| | NALM-16 | 882.1 ± 25.2 | 0.0683 ± 0.0178 | 0.49 ± 0.02 |
| | REH | 51.3 ± 3.5 | 0.0313 ± 0.0232 | 1.23 ± 0.04 |
| | ROS-50 | 785.9 ± 109.8 | 0.9071 ± 0.3114 | 0.38 ± 0.04 |
| | RS4;11 | 16.3 ± 0.6 | 0.3750 ± 0.1444 | 0.65 ± 0.12 |
| | SEM | 64.1 ± 2.6 | 0.3203 ± 0.1840 | 0.08 ± 0.03 |
| | TANOUE | 287.8 ± 30.3 | 0.1095 ± 0.0574 | 0.14 ± 0.03 |
| | TOM-1 | 9.6 ± 0.2 | 0.0041 ± 0.0019 | 0.47 ± 0.03 |
