# Supplementary material for: Differences between intrinsic and acquired nucleoside analogue resistance in acute myeloid leukaemia cells
Source: J Exp Clin Cancer Res. 2021 Oct 12;40:317. doi: 10.1186/s13046-021-02093-4 (PMC8507139; doi:10.1186/s13046-021-02093-4)
Supplement: Supplementary file 12 — Additional file 12: Supplementary Table 3. Relative Caspase 3/7 activity in CRISPR–Cas9-mediated SAMHD1 knockout THP-1 cells (THP-1 KO) or control cells (THP-1 CTRL) following treatment with CNDAC for 24, 48, or 72 h, shown as fold-change compared to the respective untreated control. [file 13046_2021_2093_MOESM12_ESM.pdf]

Supplementary Table 3. Relative Caspase 3/7 activity in CRISPR–Cas9-mediated SAMHD1 knockout THP-1 cells (THP-1 KO) or control cells (THP-1 CTRL) following treatment with CNDAC for 24, 48, or 72 hours, shown as fold-change compared to the respective untreated control.

	CNDAC [µM]	Relative Caspase 3/7 activity [fold change to untreated]	
		THP-1 CTRL	THP-1 KO
24h	0.015	1.05 ± 0.12	1.11 ± 0.06
	0.94	0.78 ± 0.06	1.06 ± 0.16
	60	0.84 ± 0.02	3.23 ± 0.31
48h	0.015	1.20 ± 0.17	1.12 ± 0.10
	0.94	1.34 ± 0.21	2.31 ± 0.06
	60	1.47 ± 0.26	7.30 ± 0.16
72h	0.015	1.20 ± 0.18	1.36 ± 0.07
	0.94	2.16 ± 0.07	3.02 ± 0.11
	60	3.20 ± 0.23	6.60 ± 0.21
