# Supplementary material for: Differences between intrinsic and acquired nucleoside analogue resistance in acute myeloid leukaemia cells
Source: J Exp Clin Cancer Res. 2021 Oct 12;40:317. doi: 10.1186/s13046-021-02093-4 (PMC8507139; doi:10.1186/s13046-021-02093-4)
Supplement: Supplementary file 14 — Additional file 14: Supplementary Table 5. Data collection and refinement statistics for structure of SAMHD1 HD bound to CNDAC-triphosphate. [file 13046_2021_2093_MOESM14_ESM.pdf]

Supplementary Table 5. Data collection and refinement statistics for structure of SAMHD1 HD bound to CNDAC-triphosphate.

	CNDAC-TP
**Data Collection**	
Wavelength (Å)	0.979
Space Group	$P\ 1\ 2_1\ 1$
Cell dimensions	
a, b, c (Å)	85.8, 146.2, 99.6
α, β, γ (°)	90, 114.3, 90
Molecules/asymmetric unit	4
Resolution (Å)	50-2.8 (2.9-2.8)
Unique reflections	51436 (3706)
R_{merge}	0.095 (0.659)
I/σ	11.6 (1.0)
Completeness (%)	93.4 (67.9)
Redundancy	3.3 (2.2)
CC½	0.99 (0.53)
**Refinement**	
Number of non-hydrogen atoms	16342
R_{work}/R_{free} (%)	21.1/24.6 (38.1/42.6)
Average B factor	36.6
RMSD	
Bond lengths	0.02
Bond angles	1.97
Ramachandran Analysis	
Preferred regions (%)	98.1
Allowed regions (%)	1.8
Outliers (%)	0.1

Statistics in parentheses indicate those for highest resolution shell.
